# Article Watch, August 2026

**DOI:** 10.7171/001c.167624

**Published:** 2026-09-01

**Authors:** Clive A Slaughter

**Affiliations:** 1 Department of Interdisciplinary Biomedical Sciences UGA School of Medicine

**Keywords:** N/A

## Abstract

This column highlights recently published articles that are of interest to the readership of this publication. We encourage ABRF members to forward information on articles they feel are important and useful to Clive Slaughter, UGA School of Medicine, Department of Interdisciplinary Biomedical Sciences, 1425 Prince Avenue, Athens GA 30606. Email; cslaught@uga.edu or to any member of the editorial board. Article summaries reflect the reviewer’s opinions and not necessarily those of the Association.

## MASS SPECTROMETRY

**Wright S, Cassidy N, Colburn A and O’ Connor P B**. **Chromatography-free analysis of mixtures using a two-dimensional mass spectrometry (2DMS)-enabled quadrupole time-of-flight (QToF) analyzer**. ***Analytical Chemistry*98**; **2026**: **12883–12894**. **DOI: 10.1021/acs.analchem.6c00486**

In two-dimensional mass spectrometry (2DMS), the components of a mixture of precursor ions are fragmented simultaneously. The product ion intensities are represented by peaks on a 2-D surface at positions that represent their own *m/z* value on the x-coordinate and the *m/z* value of the precursor on the y-coordinate so that all fragments may be associated with their precursors of origin. This technique was originally formulated for Fourier transform ion cyclotron resonance (FTICR) mass spectrometers to analyze complex mixtures without the need to separate the mixture components by chromatography. Wright et al. here demonstrate adaptations of the technique on a quadrupole time-of-flight (QToF) mass spectrometer to show that it can be implemented on a high-resolution system of lower cost and complexity than FTICR. The QToF quadrupole is converted to a linear ion trap in which the orbital radii of the trapped ions are modulated incrementally using resonant dipolar excitation at the characteristic frequencies of ion motion within the trap to excite ions for fragmentation. For this purpose, the authors use a modulation protocol known as stored waveform ion radius modulation (SWIM). The protocol is adapted for either ultraviolet photodissociation (UVPD) at 213 nm or collision-induced dissociation (CID). For UVPD, a Gaussian laser beam is directed along the central axis of the ion trap, and ions following a low orbital radius are fragmented where they encounter the beam’s maximal energy. For CID, ions excited to a higher radius have more kinetic energy and are therefore fragmented preferentially by collision with residual gas in the trap. The authors also explore an alternate fragmentation pathway in which ions at high excitation amplitude are transmitted into a downstream collision cell for CID. The methodology is demonstrated in a proof-of-principle analysis of a mixture of 4 peptides (polymyxin, melittin, bacitracin, and substance P). It is hoped that the procedure will obviate the need for chromatographic resolution of peptide mixtures in suitable cases.

**Afroz R, Bowen K P, Liu W and Gadkari V V**. **Native charge detection mass spectrometry of kilobase-scale messenger RNAs**. ***Analytical Chemistry*98**; **2026**: **16152–16162**. **DOI: 10.1021/acs.analchem.6c00192**

The therapeutic application of mRNAs has created a need for detailed characterization of mRNAs destined for administration to patients. Afroz et al. document the capabilities of mass spectrometry to supply structural information for such quality control. The mass of mRNA molecules may be high, their structure may be heterogenous, and their polyanionic character produces abundant cation adduction. These circumstances together render conventional electrospray ionization (ESI) mass spectrometry problematic because multiple charge states of these complex, megadalton ion populations may be impossible to deconvolute. The authors therefore investigate charge detection mass spectrometry (CDMS), which helps overcome many of the limitations of ESI by yielding values for both the *m/z* and the charge-state (*z*) for individual ions. Using a Q Exactive Ultra-High Mass Range (UHMR) Hybrid Quadrupole Orbitrap mass spectrometer from Thermo Fisher Scientific (San Jose, CA, USA) with direct mass capability, they analyze mRNAs of 997–4522 nucleotides (calculated masses of 322.25–1465.53 kDa). Under native conditions and gentle transmission, CDMS resolves mRNA impurities that differ from the intended structure by only a few nucleotides and noncovalent interactions between mRNA molecules. However, mass values are shifted ~3–11% by cation adduction. High energy collisional dissociation (HCD) improves mass spectral resolution and improves mass accuracy to within ~1–2% of calculated mass for the smaller mRNAs, but for the largest mRNA, a mass offset of +7% persists. Denaturation of mRNAs in 50% methanol further reduces adduction to provide a mass offset of 5% for the largest mRNA, but it additionally produces unhelpfully higher charge states and new oligomerization. Combining denaturation and HCD brings the mass offset down to +1.8%. CDMS in negative ion mode yields slightly higher adduction than positive ion CDMS. Mass spectrometric characterization of large mRNAs remains an intensely difficult application. The authors’ study reveals persistent mass-dependent limitations to the accuracy of the methodology.

## METABOLOMICS

Hambidge T, Corless S, Cowen S, Short M, Hopley C and Sears P. Pitfalls and inherent biases in liquid handling robotics: Investigations in automation for SI traceable measurements. *Analytical Chemistry* 98; 2026: 16107–16116. DOI: 10.1021/acs.analchem.6c00078

Automated liquid handling is employed extensively in high-throughput and screening laboratories to improve productivity and reduce error. The present study evaluates the accuracy of robotic systems that use syringe-based robotics for automated sample preparation in multianalyte quantification and assesses biases these systems may introduce. Two syringe-based robotic systems are studied: the 7696A Sample WorkBench from Agilent Technologies (Santa Clara, CA, USA) and the MultiPurpose Sampler from GERSTEL (Mülheim, Germany). Both incorporate integrated balances for gravimetric measurements of liquid delivery. Amino acids obtained as certified reference materials from the National Institute of Standards and Technology (NIST) are quantified using gas chromatography-mass spectrometry in a double isotope dilution mass spectrometric workflow. The results are compared with those obtained by manual sample preparation. The authors detect various sources of bias, both expected and unexpected. Fluid evaporation occurs from uncapped vials, and acetonitrile evaporates faster than water. Accuracy of fluid delivery is affected by recipient vial caps, by delivery needle placement, and by draw and dispensing speeds. However, when automated conditions are optimized, bias is greatly reduced, and measurement uncertainty becomes comparable to manual preparation. This study alerts investigators to errors that may emanate from the use of automated systems for sample preparation, provides means to detect such errors, and suggests ways to mitigate their effects.

## MACROMOLECULAR CHARACTERIZATION

**Petrov P N, Zhang J T, Remis J, Axelrod J J, Cheng H, Cooper E S, Hicklin I K, Sandhaus S, Schnurr C, Glaeser R M and Müller H**. **Laser phase plate improves structure determination of small proteins by cryo-EM**. ***Science*393; 2026: 195-199**. **DOI:10.1126/science.aeh0665**

Cryo-electron microscopy (cryo-EM) has contributed greatly to solution of three-dimensional protein structures, most especially for the numerous proteins that are hard to crystallize for X-ray diffraction. However, cryo-EM has hitherto proved unable to provide requisite information for proteins <50 kD. Recognizing that the electron wave function is minimally absorbed by proteins and is poorly phase-shifted, Petrov et al. have explored methods for improving contrast in protein images by introducing phase plates into the electron beam so that they can perform phase contrast EM. Introduction of material phase plates has proven unsatisfactory, but the authors here describe proof-of-principle experiments indicating that a laser “phase plate” may contribute substantially to resolution of this limitation. An extremely high-intensity laser beam of continuous light intensity of ~400 GW/cm^2^ is focused upon the electron beam to induce a phase shift of 88±7° by stimulated Compton scattering. This capability is incorporated into a purpose-built Krios G4 electron microscope from Thermo Fisher Scientific, equipped with a CEOS CETCOR spherical aberration corrector. The authors use this system for structural analysis of aldolase (157 kDa) and, more challengingly, hemoglobin (64 kDa). They collect laser-on and laser-off data for comparison. The laser improves resolution, apparently through improved ability to correct for motion of the micrographs, through improved identification of particles and assessment of their orientation on the substrate, and through better utilization of information from the early frames in image collection when the sample has incurred least radiation damage. The authors view these results as preliminary and hope to pursue imaging under improved conditions, which include replacement of defocused imaging, performed here for enhancement of contrast, with imaging in-focus to enhance high-frequency spatial frequency information.

## PROTEOMICS

**Al Siblani S, Armengaud J and Lozano C**. **Systematic evaluation of depletion and enrichment technologies for platelet-free plasma proteomics**. ***Journal of Proteome Research*25**; **2026**: **2723–2739**. **DOI:10.1021/acs.jproteome.5c01056**

**Kverneland A H, Østergaard O, Schmidt L, Østergaard Johansen M, Ullitz Thorsen S, Frikke Schmidt R, Christoffersen C and Olsen J V**. **Benchmarking plasma proteomics workflows and their correlation to clinical routine protein assays**. ***Journal of Proteome Research*25**; **2026**: **3188–3200**. **DOI:10.1021/acs.jproteome.6c00078**

**Völlmy F, Kaushik P, Eckert S, Paschen C, Tavalaei S, Fidelin J, Weiser S and Bantscheff M**. **Comparison of common plasma proteomics workflows reveals distinct preanalytical biases by sample preparation and nanoparticle enrichment**. ***Journal of Proteome Research*25**; **2026**: **3178–3187. DOI:10.1021/acs.jproteome.6c00048**

Proteomic analysis of blood plasma based upon combined liquid chromatography-mass spectrometry (LC-MS) is believed to afford exceptional potential for disease biomarker discovery because the dynamic range achieved by this methodology is impressively high — some 3 orders of magnitude. However, realization of this potential is compromised by the still much greater range of abundance of plasma proteins —12 orders of magnitude. The discrepancy is somewhat narrowed by numerous new techniques involving selective depletion of the most abundant proteins or enrichment of the less abundant ones. Three studies here compare the effectiveness of various examples of these new techniques in terms of proteome depth, reproducibility, linearity, and quantitative precision. A finding shared by all three studies is that contaminating cells and extracellular vesicles of hematopoietic origin — platelets, erythrocytes, and mononuclear cells — may contribute substantially to the complexity of the plasma proteome. This finding focuses attention on the centrifugation protocol used to generate plasma samples, indicating the importance of high-speed centrifugation to eliminate contaminants of cellular origin. This step is particularly important with depletion techniques, which are more sensitive to the presence of contaminants.

**Pomogaev D D, Gorshkov M V and Ivanov M V**. **Nature of false peptide identifications in data-independent acquisition-based proteome analysis**. ***Journal of the American Society for Mass Spectrometry*37**; **2026**: **1333–1341**. **DOI:10.1021/jasms.5c00320**

In proteomic analyses performed with liquid chromatography-tandem mass spectrometry (LC-MS/MS), peptide identification based on data-independent selection of precursor ions has gained in popularity. In data-independent selection of precursor ions for fragmentation, relatively broad *m/z* isolation windows containing many different precursors are employed. Consecutive isolation windows are programmed in a cyclical manner to cover the desired range of precursor *m/z* values. In data-dependent selection, relatively narrow *m/z* isolation windows containing sometimes just one precursor are employed, chosen based on a prominent signal in a preceding precursor ion scan. The reasons for the preference for data-independent precursor selection are that the stochastic nature of precursor selection in data-dependent acquisition is avoided, and the process can be conducted rapidly if broad precursor *m/*z windows are employed. Product ions derived from different precursors may be distinguished retrospectively by criteria that include their distinct chromatographic retention times. However, uncertainty about the identification of precursors may yield false peptide identifications upon matching product ion spectra with spectral databases. Such uncertainty is the subject of the present study. The authors choose the data-independent acquisition method, DIA-NN, which uses deep neural networks for reporting peptide identifications. The authors analyze LC-MS/MS results acquired using the Universal Proteomics Standard, a mixture of 48 purified human proteins supplied by Sigma-Aldrich (St. Louis, MO, USA) mixed with the *E. coli* proteome at known concentrations. For data-independent acquisition, they employ isolation windows of width 15 Th and 7.5 Th. They search several hundred artificially generated databases that contain sequences for the human target proteins incorporating amino acid replacements (all of which produce substantial mass shifts) that mimic single amino acid substitutions. False identifications using these databases is based on similar fragmentation patterns. The incidence of such false identifications is shown to significantly exceed false discovery rates based on target-decoy calculations with shuffled sequences. For example, up to 21% of the identifications based on the true database are still reported when the second amino acid in peptides is substituted. The number decreases with distance from the *N*-terminus. This observation is linked to the lower intensity of *b*-ion (N-terminal fragment) signals than that of *y*-ion (C-terminal fragment) signals in product ion spectra produced by collision-induced fragmentation. An average of 40% fewer incorrect matches is seen with the narrow isolation window than with the broad one. Encouragingly, when correct sequences are contained in the database as well as incorrect sequences, incorrect matches are further reduced by 3x. The excess of incorrect matches is essentially eliminated when searching datasets acquired by data-dependent scanning. Nevertheless, these results highlight the importance of utilizing correct precursor ion mass values in peptide identification. The work suggests how the accuracy of peptide identification strategies might be reassessed.

## FUNCTIONAL GENOMICS & PROTEOMICS

**Jerabek S, Kim J, Sung J, Jung C, Kulmann M I R, Isado M, Jang H-S, Li M, Bhatele S, Kappy M, Xu S, Hwang G-H, Xu J, Marin D, Woo J-S, Bae S, Treff N and Egli D**. **Efficient base editing and development in human embryos without chromosomal alterations**. ***bioRxiv*2026**: **2026.05.30.728989**. **DOI:10.64898/2026.05.30.728989**

In a preprint prior to review, Jerabek et al. describe methodology that significantly improves technical capability for germline editing of human embryos. Germline editing using CRISPR/Cas9 editing is recognized to be unsuitable for such editing because it induces a high incidence of undesired chromosome breaks, aneuploidy, chromosomal rearrangements, deletions, and sequence alterations. These detrimental outcomes are associated with the double-stranded breaks in DNA upon which CRISPR/Cas9 relies to accomplish intended sequence alterations. Base editing relies instead on single stranded breaks. Consequently, greater precision and efficiency is anticipated. In the present study, MII oocytes donated for fertilization and fertilized oocytes stored for fertility treatment are employed for editing following consensual donation for research. The loci chosen for base editing are *PCSK9*, which regulates levels of plasma low density lipoprotein, and *HBG1/2*, which mediate hemoglobin F expression. The authors discover that when the base editor is delivered to recipient cells by injection of the encoding mRNA at a single cell stage, along with the guide RNA, it causes developmental arrest prior to blastocyst formation. However, when the base editor is delivered as a translated protein, it allows normal development. The authors further demonstrate precision and efficiency of editing, and show absence of large deletions and aneuploidy, although short insertions adjacent to targeted bases within the editing window at *HBG1/2* are observed at low frequency. These results represent a significant technical improvement, but they do not imply that the technology is ready for clinical implementation. The work also does not address ethical issues surrounding decisions on undertaking embryo editing for experimental or therapeutic purposes and does not consider what criteria might be used for selection of gene targets for editing.

**Sanderson E, Levin M G, Walker V, Yuan S, Badini I, Dolce J, Mahida K J, Nho J-W, Pingault J-B, Damrauer S M, Hemani G and Davies N M**. **Challenges and future directions for Mendelian randomization**. ***Nature Genetics*58**; **2026**: **984–994**. **DOI:10.1038/s41588-026-02546-6**

Mendelian randomization is a statistical tool to test whether a phenotypic trait or process associated with a disease actually causes the disease or is simply associated with it through some common cause. For example, low plasma levels of high-density lipoprotein (HDL) — the “exposure” in the test — are associated with coronary artery disease (CAD) — the “outcome.” But, is HDL protective? Alleles at various gene loci are associated with HDL level but not with the levels of other lipids known to be causally associated with CAD (such as low-density lipoprotein, LDL). The frequency of these alleles may be used as “instrumental variables” in Mendelian randomization testing because it is reasonable to suppose that the only effect they could have on CAD is through HDL level. Recent studies show that the correlation between CAD and the incidence of these alleles is inconsistent, indicating that HDL is not protective. This conclusion is now supported by mechanistic studies. The association between CAD and low HDL probably results from the association of both with type 2 diabetes mellitus. The internal validity of such Mendelian randomization tests relies on three assumptions. Firstly, the “instrumental variables” must be relevant to the “exposure.” Secondly, the “instrumental variables” must be independent of the “outcome” in the sense that there is no common cause affecting both (e.g., sampling bias). Thirdly, the “instrumental variables” can only influence the “outcome” through the “exposure.” This may be the most difficult requirement to satisfy, owing to the possible existence of unknown pleiotropic effects of the genetic variants. These requirements for internal validity of Mendelian randomization are often difficult to establish. The authors describe statistical means of checking them. Nonetheless, they emphasize the ultimate importance of molecular, cellular, animal, and experimental studies to assess the validity of the assumptions underlying any Mendelian randomization test, and the causal inferences deduced from it, exemplified by the mechanistic studies in the case of HDL.

**Gerritsen M, Ray P S, Sekaran T, Schwarzl T, Hentze M W and Kulozik A E**. **Cross-contamination of commercial oligonucleotides with library-structured sequences**. ***Nucleic Acids Research*54**; **2026**: **gkag433. DOI: 10.1093/nar/gkag433**

Gerritsen et al. relate an experience in which results from a high-throughput sequencing experiment that proved uninterpretable could be traced to inadvertent contamination of custom-synthesized index adaptor oligonucleotides. Many high-throughput sequencing procedures, including bulk RNA-seq, ChIP-seq, ATAC-seq. and DNA methylation profiling, among many others, rely on adaptor and index oligonucleotides for library preparation. They depend critically upon oligonucleotide purity. The present authors provide a quality control strategy for identifying and tracing contaminants derived from oligonucleotides to be used for preparation of high-throughput sequencing libraries. It involves mapping all sequencing reads to the genome of interest, identifying the origin of all unmapped reads by searching sequence databases, and directly testing the purity and sequence integrity of supplied oligonucleotides.

## CELL BIOLOGY

**Liu H, Noguera-Ortega E, Dong X, Lee W D, Chang J, Aydin S A, Li Y, Shin Y, Shi X, Liousia M, Martinez M C, Brotman J J, Kim S, Chen Z, Wang A, Ou Z, Paek J, Park J Y, Liu A, Hu H, Xiao Z, Racca D M, Kim S-j, Worthen G S, Guo W, Puré E, Kang T, Rabinowitz J D, Wherry E J, Moon E K, Albelda S M and Huh D D**. **A tumor-on-a-chip for in vitro study of CAR-T cell immunotherapy in solid tumors**. ***Nature Biotechnology*44**; **2026**: **959–975**. **DOI:10.1038/s41587-025-02845-z**

**Maulana T I, Teufel C, Cipriano M, Roosz J, Lazarevski L, van den Hil F E, Scheller L, Orlova V, Koch A, Hudecek M, Alb M and Loskill P**. **Breast cancer-on-chip for patient-specific efficacy and safety testing of CAR-T cells**. ***Cell Stem Cell*31**; **2024**: **989–1002.e9**. **DOI: 10.1016/j.stem.2024.04.018**

Two microfluidic studies describe new in vitro culture models of solid tumors that may be used for characterizing interactions between CAR-T cells and the tumor microenvironment. The aim is to improve outcomes of immune cell therapy for solid tumors. The innovation in both studies is to provide endothelial cells that generate structures emulating the vascular bed supplying the tumor. This allows investigation of extravasation of CAR T-cells, cell signaling between tumor and CAR-T cells, and the immunosuppressive microenvironment of the tumor. The microfluidic devices place tumor explants and stromal cells in a channel that communicates with a separate hydrogel-filled channel(s) in which endothelial cells form functional vascular structures through which CAR-T cells may be introduced. Liu et al. utilize human lung adenocarcinoma tumors and malignant pleural mesothelioma. They deploy human umbilical vein endothelial cells or primary human lung microvascular endothelial cells for vascularization. Maulana et al. use a breast cancer cell line or organoids made from patient-derived primary breast cancer cells. They derive microvascular endothelial cells from skin. The experiments of both groups are preliminary in nature. The model endothelial cells may not faithfully emulate the behavior of tumor vasculature. However, experimental systems of these types foreshadow capabilities to conduct controlled studies of the nature of cytokine signaling and pharmaceutical interventions to optimize CAR-T cell therapy for solid malignancies.

## IMAGING

**Ochner H, Isbilir B, Blasche S, Scheidweiler D, Zhang Y, Wang Z, Smith T, Franco C, Bradley R, Patil K R and Bharat T A M**. **Subcellular chemical mapping using correlated cryogenic electron and mass spectrometry imaging**. ***Nature Methods*23**; **2026**: **1174–1183**. **DOI:10.1038/s41592-026-03109-7**

**Taubitz T, De Castro O, Andersen D, Berro Z, Hans S, Tabean S, Wachsmuth-Melm M, Hobler G, Nelissen I, Lucas F, Eswara S, Chlanda P, Wirtz T, Audinot J-N and Biesemeier A**. **Cryo-HIM-SIMS on the npSCOPE: Correlative topographic, transmitted and SIMS imaging at cryogenic temperatures**. ***Analytical Chemistry*98**; **2026**: **12317–12327**. **DOI:10.1021/acs.analchem.5c06908**

The recent successful demonstration of methods for chemical analysis of vitrified materials by secondary ion mass spectrometry (SIMS) using a focused beam of primary ions opens the possibility of combining this capability with high resolution imaging of the same specimen by cryo-electron microscopy (cryo-EM) or a focused beam of suitable alternate charged particles. Two groups make progress in implementing such combined technology. Ochner et al. image specimens by cryo-EM and then transfer them to a cryo-SIMS instrument equipped with a time-of-flight mass analyzer. They use a focused beam of gallium ions to desorb molecular ions from the specimen surface. They achieve a spatial resolution of 50 nm in SIMS analysis. The authors study the subcellular distribution of the fluorinated environmental contaminant bisphenol-AF within bacterial cells that take it up. Taubitz et al. perform secondary electron imaging in which electrons ejected by a focused primary ion beam of helium or neon ions are monitored. Chemical analysis uses the same ion beam to eject molecular ions from the specimen for SIMS. In this implementation, a double focusing magnetic sector mass analyzer is used for SIMS. These authors achieve a spatial resolution of 10 nm in SIMS. They also pay special attention to the design of the sample stage and conditions for transfer of specimens into the instrument to avoid artefactual structural changes or contamination. The implementation of both groups restricts the detectable mass range to <400 amu, but this mass range is amenable to extension by using a primary ion beam of larger particles.

## DESIGN & DEVELOPMENT OF THERAPEUTICS

**Anketell M J, Fung E, Liu W, Shinde M, He C Q, Ng K W C, Silverman S M, Campeau L-C, Pantophlet R and Britton R**. **A unified platform for nucleoside analog synthesis**. ***Science*392**; **2026**: **eaed6880**. **DOI: 10.1126/science.aed6880**

Nucleoside analogs constitute a major class of drugs that have found particularly important roles in the treatment of viral infection and cancer. The synthesis of these drugs has conventionally been performed in a laborious series of reactions to modify a chiral carbohydrate (e.g. D-ribose) followed by the attachment of a nucleobase via a nucleophilic nitrogen to yield an *N*-linked nucleoside. *C*-linked nucleosides can also be synthesized using organometallic nucleobase derivatives. However, none of the available methods lend themselves to the parallel production of diverse species in large numbers to form the extensive compound libraries that best exploit modern, high-throughput screening methods for discovery of new therapeutics. Anketell et al. discover a single building block that can be readily prepared in enantiomeric purity from cheap, achiral materials — an *N*-methyliminodiacetyl boronate. From this building block, they prepare by simple pathways iminonucleosides and thionucleosides as well as ribonucleosides. They synthesize both *N*-linked and *C*-linked nucleosides, C4′-modified nucleosides, and ProTides (phosphate pro-drugs). Using their methodology, they generate a diverse, 71-member library for high-throughput screening. With this library, they identify 3 new compounds with antiviral activity in the nanomolar to micromolar range.

